# Supplementary material for: Dysregulation of Glycerophosphocholines in the Cutaneous Lesion Caused by Leishmania major in Experimental Murine Models
Source: Pathogens. 2021 May 13;10(5):593. doi: 10.3390/pathogens10050593 (PMC8152770; doi:10.3390/pathogens10050593)
Supplement: Supplementary file 1 [file pathogens-10-00593-s001.zip › supplementary material/supplementary figure 2.pdf]

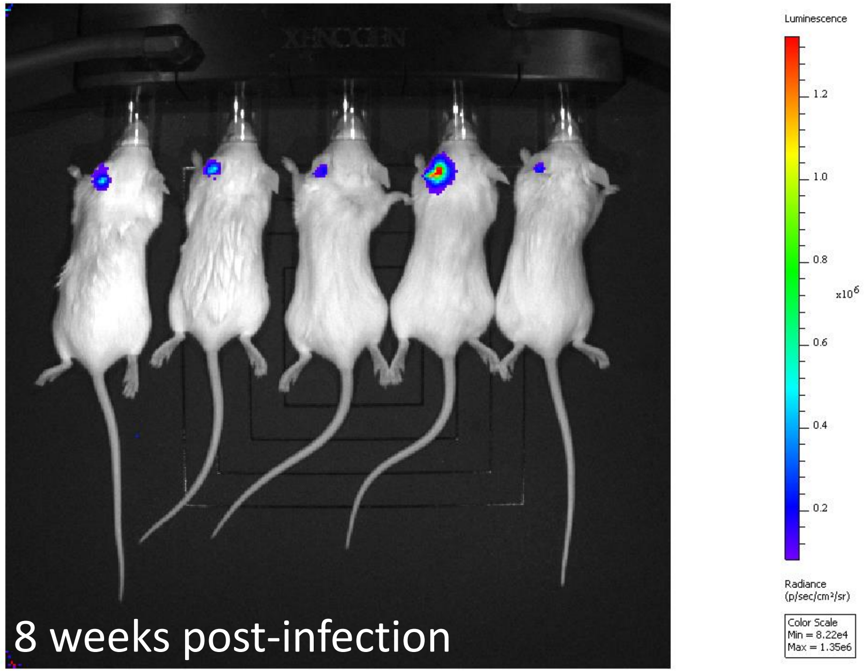

Mouse number	Luminescence counts
mouse 1	2.18E+04
mouse 2	1.84E+04
mouse 3	1.02E+04
mouse 4	7.95E+04
mouse 5	7.35E+03

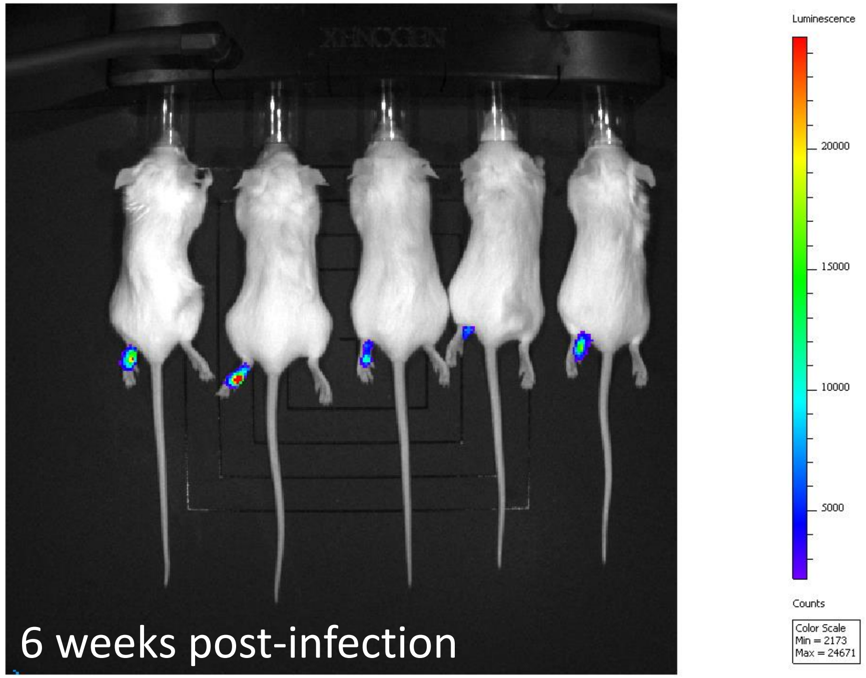

Mouse number	Luminescence counts
mouse 1	5.11E+05
mouse 2	6.46E+05
mouse 3	2.41E+05
mouse 4	1.44E+05
mouse 5	4.13E+05
