# Supplementary figures and images for: Dysregulation of Glycerophosphocholines in the Cutaneous Lesion Caused by Leishmania major in Experimental Murine Models
Source: Pathogens. 2021 May 13;10(5):593. doi: 10.3390/pathogens10050593 (PMC8152770; doi:10.3390/pathogens10050593)

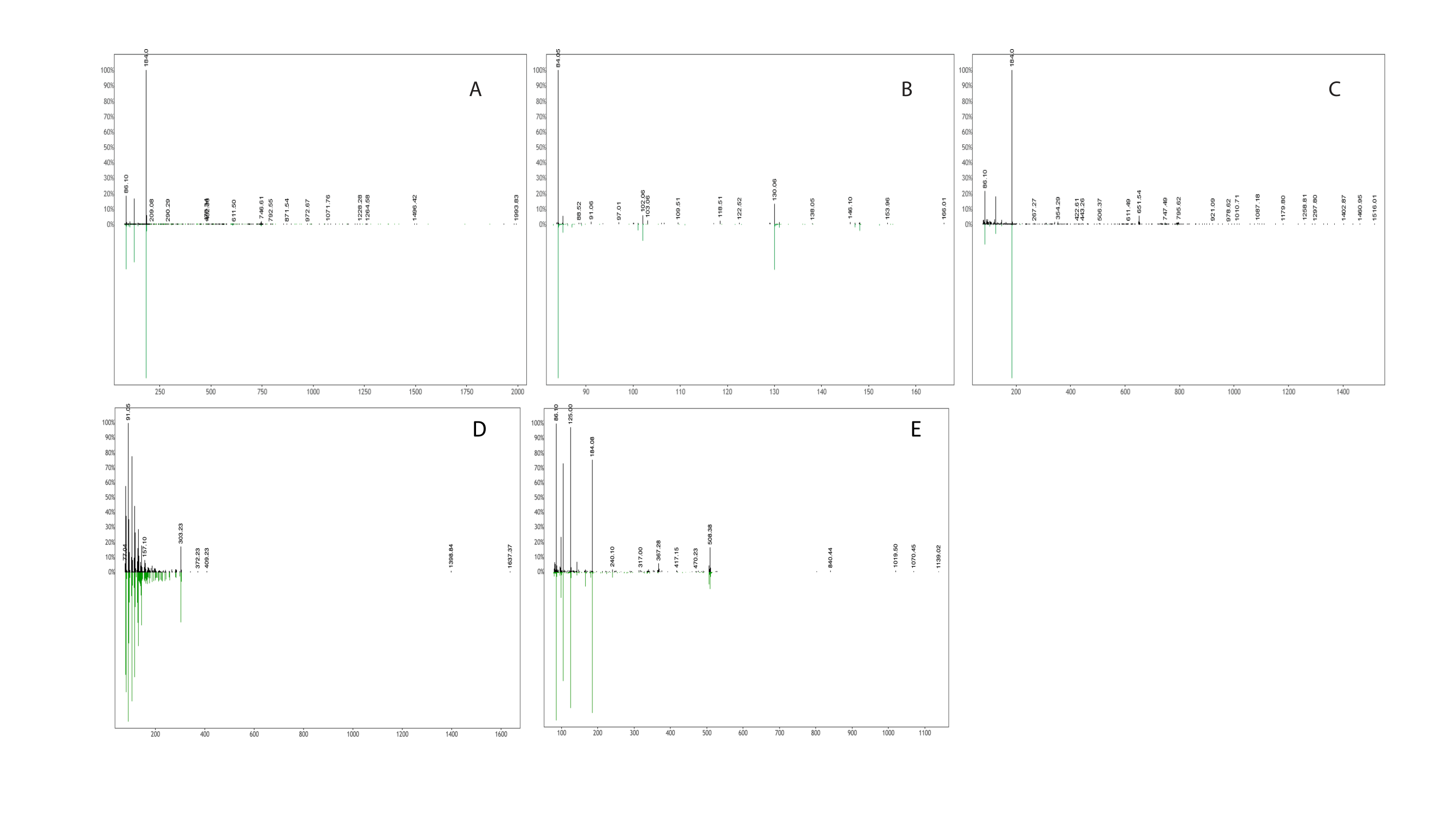

Supplement: Supplementary file 1 [file pathogens-10-00593-s001.zip › supplementary material/supplementary figure 1.tiff]
